# When to use social information: the advantage of large group size in individual decision making

**DOI:** 10.1098/rsbl.2007.0017

**Published:** 2007-02-06

**Authors:** Andrew J King, Guy Cowlishaw

**Affiliations:** 1 Institute of Zoology, Zoological Society of LondonRegent's Park, NW1 4RY London, UK; 2 Department of Anthropology, University College LondonTaviton Street, London WC1H 0BW, UK

**Keywords:** pooling information, social information, personal information, group size

## Abstract

Correct decision making is crucial for animals to maximize foraging success and minimize predation risk. Group-living animals can make such decisions by using their own personal information or by pooling information with other group members (i.e. social information). Here, we investigate how individuals might best balance their use of personal and social information. We use a simple modelling approach in which individual decisions based upon social information are more likely to be correct when more individuals are involved and their personal information is more accurate. Our model predicts that when the personal information of group members is poor (accurate less than half the time), individuals should avoid pooling information. In contrast, when personal information is reliable (accurate at least half the time), individuals should use personal information less often and social information more often, and this effect should grow stronger in larger groups. One implication of this pattern is that social information allows less well-informed members of large groups to reach a correct decision with the same probability as more well-informed members of small groups. Thus, animals in larger groups may be able to minimize the costs of collecting personal information without impairing their ability to make correct decisions.

## 1. Introduction

When moving through its habitat, a group-living animal directly interacts with its environment to gather both ‘personal information’ from environmental cues and ‘social information’ from the behaviour of conspecifics (Grocott 2003; Dall *et al*. 2005). Other group members will present social information to an individual in a variety of ways (Bradbury & Vehrencamp 1998; McGregor 2005) that can be broadly categorized as evolved ‘signals’ and social ‘cues’ (Danchin *et al*. 2004; Dall *et al*. 2005). Signals refer to intentional communication while cues refer to information produced incidentally by individuals (Valone 1989; Danchin *et al*. 2004), e.g. the foraging behaviour of others reveals the location of food while flight behaviour indicates impending danger.

Individuals monitoring the behaviour of other group members may be able to make faster, more accurate assessments of their environment through the information that signals and cues provide (Valone & Templeton 2002). Similarly, theoretical work that focuses on group decision making suggests that decisions based on information pooled from many group members may be more accurate than decisions based on the information of a single individual (Simons 2003) and correct group decisions might even occur solely on the basis of cues (Couzin *et al*. 2005).

Nevertheless, animals in groups will not use social information indiscriminately. Rather, individuals will use socially acquired and personal information according to the respective reliability of these alternative information sources (Bergen *et al*. 2004; Dall *et al*. 2005). The balance between personal and social information use is thus likely to reflect individuals adjusting their decision making to exploit the most reliable information available (Nordell & Valone 1998; Bergen *et al*. 2004). The relative quality of social versus personal information and the number of individuals sharing information (i.e. group size) are likely to be crucial determinants in this process (e.g. Bergen *et al*. 2004; Fraser *et al*. 2006). However, a general understanding of how these factors interact to influence information use remains to be established.

In this paper, we use a simple model to investigate how individuals might balance their use of personal information against social information when making decisions. Drawing on Condorcet's eighteenth century jury theorem, we first investigate how the quality of social information, i.e. personal information pooled across *n* group members, varies with both the number of individuals and the quality of the personal information involved. We then ask how individuals might best balance their use of personal and social information for groups of different sizes.

## 2. Material and Methods

We use Condorcet's binomial jury theorem (following List 2004) to explore how the quality of personal information compares to the quality of social information available to an individual. We take the quality of personal information (*I*_p_) to be the probability that the personally acquired information possessed by an individual is correct. The quality of social information (*I*_s_), the probability that the majority of the group is correct, is then calculated as follows:

$$I_{\text{s}}=\sum_{k>n/2}^{n}\left(\begin{array}{c} n\\ k \end{array}\right)I_{\text{p}}^{k}{\left(1-I_{\text{p}}\right)}^{n-k},$$

where *n* is the number of individuals in the group and *k* individuals comprise the majority (e.g. in a group of five, the majority will comprise three or more individuals). All the analyses are for odd groups sizes only, 1<*n*<51, to avoid ties (where the same number of individuals are correct and incorrect). This model considers a simplified case where (i) information is discrete (e.g. a predator is present or absent, a food patch is rich or poor), (ii) group membership is homogeneous (i.e. all group members have the same quality of personal information), and (iii) personal and social information are equally available (i.e. there are no differential costs to using either type of information).

## 3. Results

First, we examined how the quality of social information varies with both the number of individuals and the quality of the personal information involved (figure 1). We found that when the quality of personal information is high (*I*_p_>0.5), individuals that pool this information should make more correct decisions than those that do not. In contrast, when the quality of personal information is poor (*I*_p_<0.5), decisions based on pooled information are less likely to be correct than those based on personal information alone. The magnitude of each of these effects is greater in larger groups (figure 1).

We then asked how individuals might best balance their use of personal and social information for groups of different size. We found that as groups grow larger, the quality of personal information required to maintain high-quality social information is reduced (figure 2). Thus, individuals in larger groups (*n*=21) can make decisions on the basis of social information with a higher likelihood of being correct (*I*_s_=0.9) when personal information is relatively low (*I*_p_=0.64), whereas individuals in smaller groups (*n*=3) would need higher-quality personal information (*I*_p_=0.80) to achieve the same level of social information accuracy.

## 4. Discussion

It is already well established that group living can provide benefits to individual group members (Krause & Ruxton 2002). Information sharing is one of these benefits: animals in groups can base their decisions not only on their own information but also that of others (e.g. Kerth *et al*. 2006). This is beneficial because individuals which observe group mates can obtain more accurate information and thus make better-informed decisions on the basis of the most reliable information available (Bergen *et al*. 2004; Fraser *et al*. 2006).

Here, we have adopted Condorcet's jury theorem to explore how group-living animals might balance their use of personal and social information. We have found that individuals are more likely to make correct decisions when they pool the personal information of others, provided that such personal information is of good quality (correct at least half of the time). Counter-intuitively, given earlier work on information use by animals (Ward & Zahavi 1973), we have also found that when personal information is of poor quality (correct less than half the time), it is better for individuals to avoid using pooled information. Perhaps, most importantly, we have also found a clear group-size effect in the reliability of personal and social information. When personal information is poor, the likelihood that social information is correct progressively deteriorates as groups grow larger. However, when personal information is of good quality, social information allows less well-informed members of large groups to reach a correct decision with the same probability as more well-informed members of small groups.

Our approach has only considered the case where all the group members have the same quality of personal information and both personal and social information are already available. These conditions will not always be met, and further modelling studies might usefully explore the effects of variation in personal information across individuals (e.g. Reebs 2000; Franks *et al*. 2002; Couzin *et al*. 2005) and the differential costs that might be involved in the acquisition and processing of personal and social information (e.g. Dall *et al*. 2005). It would also be of interest to consider those cases where information is graded rather than discrete (Valone & Templeton 2002; Dall *et al*. 2005), such that a range of information values (rather than the dichotomous correct–incorrect alternatives used here) could be explored. Nevertheless, our study provides a simple conceptual model to understand how individuals in groups of different size might balance their use of social information with personal information.

Our model may also offer a framework for improving our understanding of the benefits of living in groups. Although the use of social information is not generally likely to promote group living by itself (Beauchamp 2004), for individuals living or interacting in groups, using social information may offer yet another advantage to group living. The group size–predator detection effect (Elgar 1989; Roberts 1996) provides a classic example. Animals in larger groups are commonly observed to scan less frequently for predators while maintaining their overall detection rate, allowing them more time for feeding (for a recent example, see Fernandez *et al*. 2003). Reduced vigilance in larger groups may result from the dilution of predation risk (Roberts 1996) or an increase in feeding competition (Beauchamp & Ruxton 2003), but our model provides an explanation for how the overall detection rate can be maintained when individual vigilance has been reduced and provides further understanding of why individuals might be willing to lower their vigilance in the first place. By pooling information, animals in larger groups can make decisions with the same accuracy as those in smaller groups even when their personal information is less accurate.

## Figures and Tables

**Figure 1:**
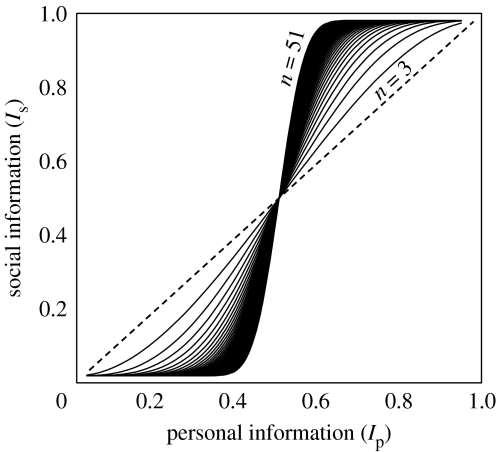
Relative quality of social versus personal information. Plot of quality of social information (*I*_s_), i.e. the probability that the majority of the group is correct, against the quality of personal information (*I*_p_), i.e. the probability that the information possessed by an individual is correct. Lines plotted are for odd group sizes (*n*) up to 51. To the left-hand side of the dashed line through the origin (*n*=1) are represented all scenarios where an individual should use social information; to the right-hand side of the line, an individual would do better to rely on its own personally acquired information.

**Figure 2:**
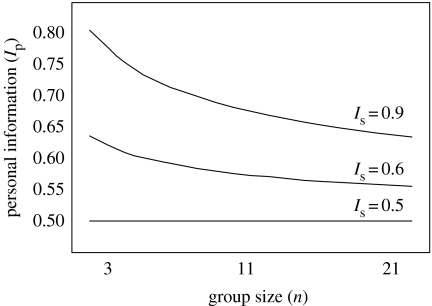
The advantage of large group size in individual decision making. Plot of quality of personal information (*I*_p_), i.e. the probability that the information possessed by an individual is correct, against group size (*n*). Lines are plotted for odd *n* (3<*n*<21), where social information (*I*_s_) is correct with probability 0.5, 0.6 and 0.9.
